# An atrial switch procedure for heart transplantation in an infant with heterotaxy-dextrocardia

**DOI:** 10.1016/j.xjtc.2021.05.003

**Published:** 2021-05-18

**Authors:** Yuki Nakamura, Adnan Al Ayoubi, Ravi Ashwath, Vernat Exil, Marco Ricci

**Affiliations:** a Division of Pediatric Cardiothoracic Surgery, University of Iowa Hospitals and Clinics, Iowa City, Iowa; b Division of Pediatric Cardiology, University of Iowa Hospitals and Clinics, Iowa City, Iowa

**Central Message**

Biatrial anastomosis with intra-atrial baffling using an atrial switch procedure may be a useful tool for heart transplantation in small pediatric patients with heterotaxy-dextrocardia.

**Figure:**
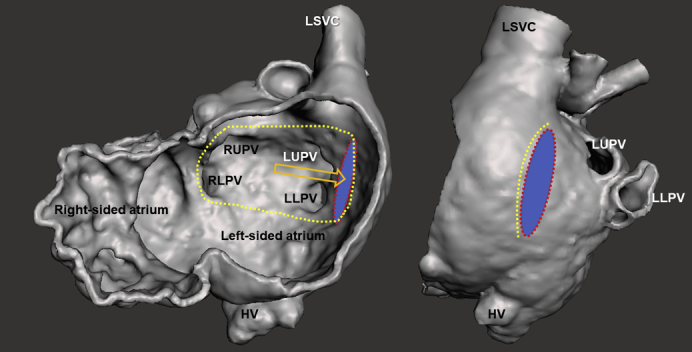
Heart transplantation in heterotaxy-dextrocardia using an atrial switch procedure.

See Commentaries on pages 192 and 194.

We report an infant with heterotaxy-dextrocardia who underwent heart transplantation using an atrial switch procedure.

## Clinical Summary

**Figure 1:**
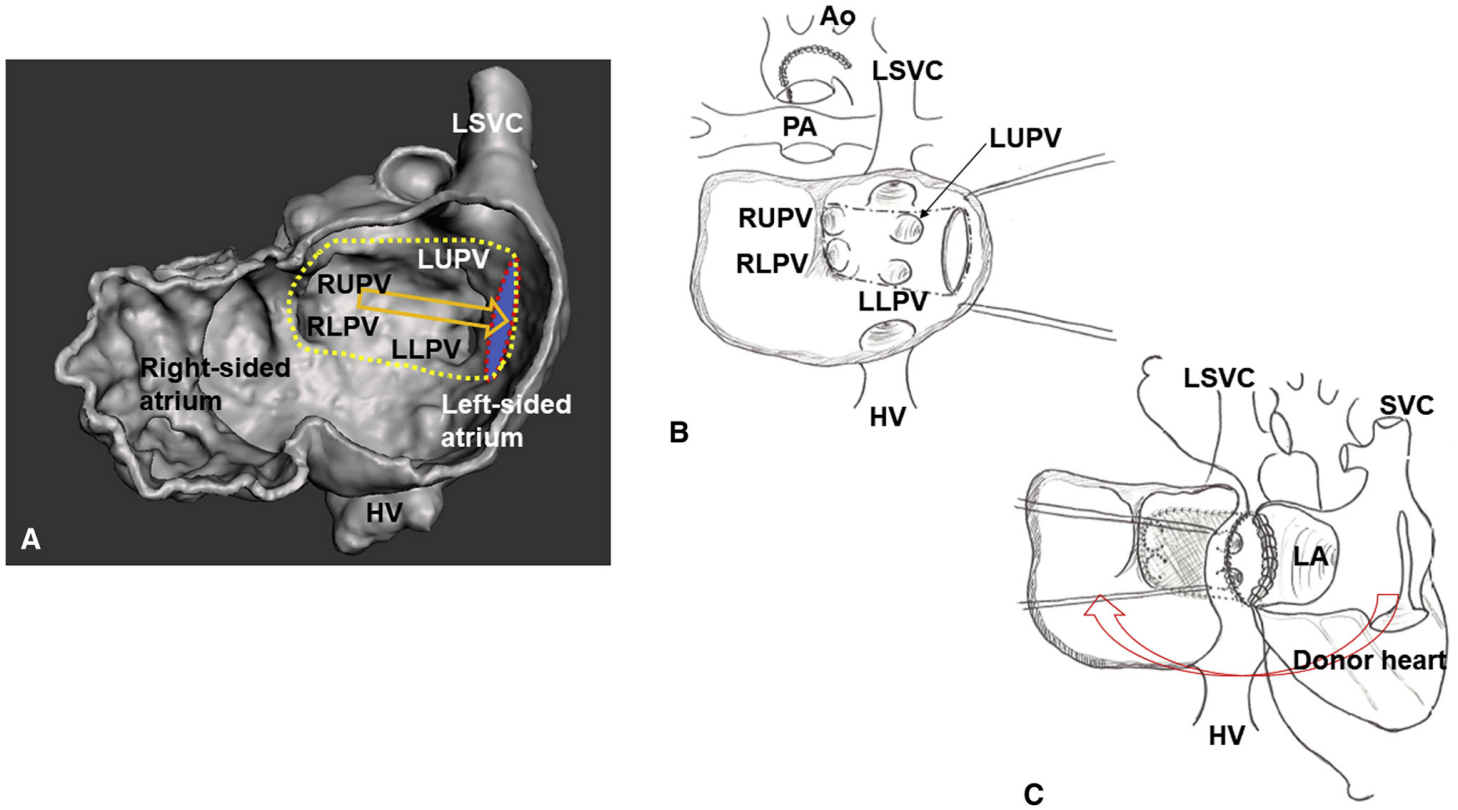
Heart transplantation in heterotaxy-dextrocardia using an atrial switch procedure is illustrated. A, A 3-dimensional image of the recipient heart reconstructed from pretransplant cardiac computed tomography angiogram is shown. The digital heart model was transected in a plane parallel to the atrioventricular groove to demonstrate inner structures of the atria. The red dotted ellipse represents an opening made in the left-sided recipient atrium anterior to the left pulmonary vein orifices. The yellow dotted line represents a suture line of a rectangular patch for the atrial switch procedure. The patch was sutured to the anterior lip of the opening to redirect pulmonary venous return (*orange arrow*). The opening (*red dotted ellipse*) was used for the left atrial anastomosis of the heart transplantation. B, An illustration of the operative field after the recipient cardiectomy is shown. The entire atrial tissue was preserved. The dotted line shows the suture line of the patch for the atrial switch procedure. C, An illustration of the operative field during the left atrial anastomosis is shown. The right atrial anastomosis was performed using a right-sided opening of the recipient atrial cuff (*red arrow*). *HV*, Hepatic veins; *LA*, left atrium (of the donor heart); *LLPV*, left lower pulmonary vein; *LSVC*, left superior vena cava; *LUPV*, left upper pulmonary vein; *RLPV*, right lower pulmonary vein; *RUPV*, right upper pulmonary vein; *SVC*, superior vena cava.

**Figure 2:**
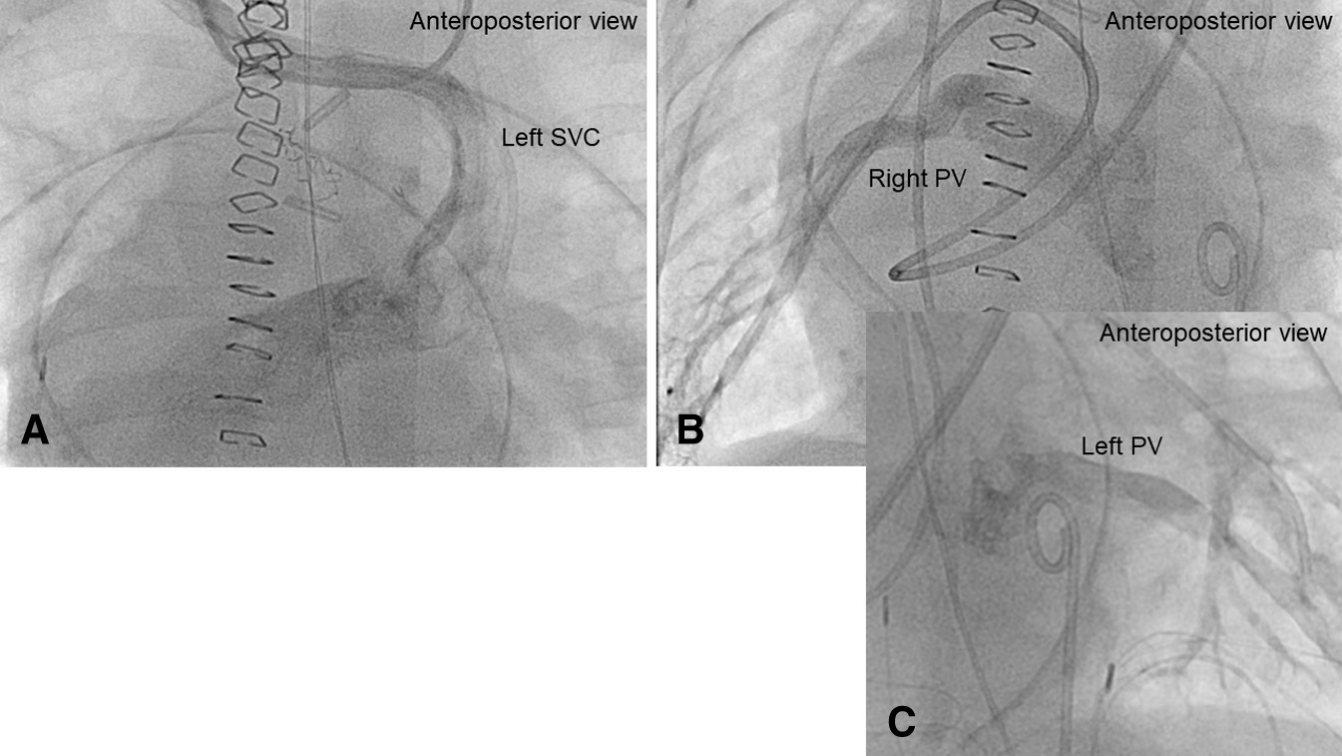
The cardiac catheterization study on posttransplant day 45 showing widely patent superior vena cava (SVC) (A), right pulmonary venous (B), and left pulmonary venous (C) pathways. *PV*, Pulmonary vein.

The institutional review board of the University of Iowa approved this report with a waiver of consent (IRB 202103110, March 11, 2021). The male patient was prenatally diagnosed with congenital heart disease. He was born at 34 weeks with a birth weight of 2364 g. A postnatal echocardiogram confirmed the diagnosis of left atrial isomerism, dextrocardia, common atrium, hypoplastic left ventricle with mitral and aortic atresia, hypoplastic aortic arch, ipsilateral pulmonary veins (PV), left-sided single superior vena cava (SVC), and interrupted left-sided inferior vena cava (IVC) with hemiazygos connection to the SVC. Both proximal coronary arteries were patent; however, systolic flow reversal was detected with coronary fistulas to the right ventricle. On day 2 of life, he developed persistent lactic acidosis with ST segment changes on electrocardiogram. An urgent bilateral pulmonary artery banding was performed to stabilize the patient on prostaglandin infusion. Cardiac catheterization and ductal stenting were planned on day 24 of life. He developed significant ST segment changes during the procedure and the intervention was aborted. Considering the atypical clinical course and persistent coronary fistulas, he was listed for a heart transplantation on day 45 of life. Ductal stenting and balloon angioplasty for banded pulmonary arteries were successfully performed at 4 months while waiting for a donor heart. At 8 months with a body weight of 8.3 kg, the patient underwent a heart transplantation using a heart from a 10.1-kg donor. The aortic arch was reconstructed using a pulmonary homograft. The recipient cardiectomy was performed preserving the entire atrial tissue. The donor heart was implanted in a levocardia position, performing an atrial switch procedure using a donor pericardial patch to develop the PV pathway (Video 1 and Figure 1). The patch was sutured starting from the right side anterior to the right PV orifices, moving forward to an opening made in the left-sided recipient atrium anterior to the left PV orifices. The left atrial anastomosis was performed using the opening. The pulmonary artery and the ascending aorta anastomoses were performed. Finally, the right atrial anastomosis was performed using a right-sided opening of the recipient atrial cuff. Weaning from cardiopulmonary bypass was smooth. Delayed sternal closure with opening of the left pericardium was performed on postoperative day 4. The patient was extubated on postoperative day 9. The cardiac catheterization study on posttransplant day 45 demonstrated widely patent anastomoses with no pressure differences in the systemic and PV pathways (Figure 2). Unfortunately, the patient died due to acute respiratory distress syndrome from human rhinovirus/enterovirus pneumonia at 4 months posttransplant after 2 weeks of venovenous extracorporeal life support. An echocardiogram at his demise showed widely patent systemic and PV pathways.

## Discussion

Surgical techniques described for heart transplantation in patients with dextrocardia/situs inversus are trichotomized: bicaval anastomosis with or without extracardiac conduits or flaps keeping levocardia,^1^ bicaval anastomosis keeping dextrocardia,^2^ and biatrial anastomosis with intraatrial baffling keeping levocardia.^3^^,^^4^ The rarity of the condition rendered previous reports small-scale with limited follow-up.

Biatrial anastomosis was adopted in our patient instead of bicaval anastomosis due to concerns over the risk of SVC pathway stenosis. Our patient was an infant who needed aortic arch reconstruction. His single left-sided SVC was short because of interrupted IVC with hemiazygos connection. Sequelae of the SVC pathway stenosis would have been greater compared with patients with bilateral SVC or without interrupted IVC.

Previous reports described biatrial anastomosis with intra-atrial baffling.^3^^,^^4^ Michler and colleagues^3^ described a technique creating 2 intra-atrial baffles using autologous atrial tissue. Our technique was unique, albeit a similar method was described by Huddleston and colleagues.^4^

There are a few technical pearls for our technique in small patients. First, the intra-atrial baffle patch cannot be redundant but has to be properly sized and rectangle-shaped to avoid systemic vein pathway obstruction. Second, the opening made on the recipient's left-sided atrium cannot be extended too far superiorly and inferiorly due to a concern over systemic vein pathway obstruction. If needed, the opening can be extended rather posteriorly. Third, during the left atrial anastomosis, the recipient's left-sided atrial wall remote from the opening (not the opening itself) can be sutured to the donor left atrium if there is a concern over the purse-string effect along the suture line in tiny patients. Fourth, as described by Boston and colleagues,^5^ choosing an appropriately sized donor is critical. We performed a similar volumetric analysis as described by them.^5^ A donor-to-recipient weight ratio was 1.2:1 in our case.

## Supplementary Data

Video 1 An intraoperative recording of a heart transplantation using an atrial switch procedure in an infant with heterotaxy-dextrocardia. Video available at: https://www.jtcvs.org/article/S2666-2507(21)00366-7/fulltext.
