# Supplementary figures and images for: An atrial switch procedure for heart transplantation in an infant with heterotaxy-dextrocardia
Source: JTCVS Tech. 2021 May 18;8:189–91. doi: 10.1016/j.xjtc.2021.05.003 (PMC8350877; doi:10.1016/j.xjtc.2021.05.003)

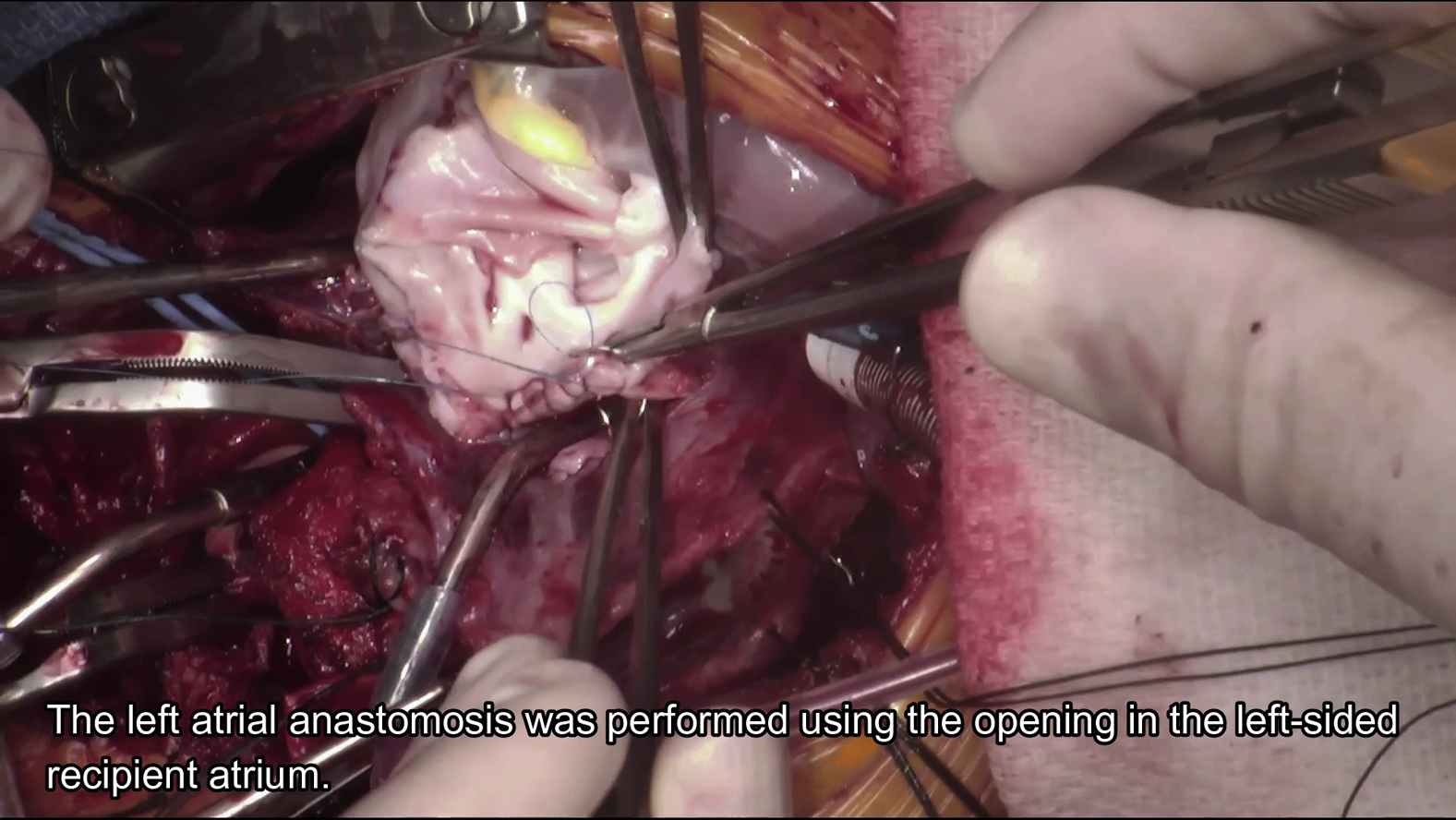

Supplement: Video 1 — An intraoperative recording of a heart transplantation using an atrial switch procedure in an infant with heterotaxy-dextrocardia. Video available at: https://www.jtcvs.org/article/S2666-2507(21)00366-7/fulltext. [file fx2.jpg]
